# In Vivo Elimination of MHC-I-Deficient Lymphocytes by Activated Natural Killer Cells Is Independent of Granzymes A and B

**DOI:** 10.1371/journal.pone.0023252

**Published:** 2011-08-11

**Authors:** Matthias Regner, Lisa Pavlinovic, Nicolie Young, Arno Müllbacher

**Affiliations:** 1 Department for Emerging Pathogens and Vaccines, John Curtin School of Medical Research, Canberra, Australia; 2 Australian National University Medical School, Canberra, Australia; MRC National Institute for Medical Research, United Kingdom

## Abstract

NK cells kill target cells mainly via exocytosis of granules containing perforin (perf) and granzymes (gzm). In vitro, gzm delivery into the target cell cytosol results in apoptosis, and induction of apoptosis is severely impaired in the absence of gzm A and B. However, their importance for in vivo cytotoxicity by cytotoxic T cells has been questioned. We used an in vivo NK cytotoxicity assay, in which splenocytes from wild-type and β_2_microglobulin-deficient (MHC-I^neg^) mice are co-injected into recipients whose NK cells were activated by virus infection or synthetic Toll-like receptor ligands. Elimination of adoptively transferred MHC-I^neg^ splenocytes was unimpaired in the absence of gzmA and gzmB, but dependent on perforin. This target cell rejection was NK cell dependent, since NK cell depletion abrogated it. Furthermore, target cell elimination in vivo was equally rapid in both wild-type and gzmAxB-deficient recipients, with the majority of specific target cells lost from lymphoid tissue within less than one to two hours after transfer. Thus, similar to T cell cytotoxicity, the contribution of gzmA and B to in vivo target cell elimination remains unresolved.

## Introduction

Gzm A and B are the most abundant and best characterized members of the granzyme family, a family of proteinases residing in the cytolytic granules of NK cells, cytotoxic T (Tc) cells and other haemopoetic cells [1]. In vitro, gzmB induces target cell death via caspase-dependent and –independent pathways, whereas gzmA mediates its effect caspase-independently (reviewed in [2]), although the cytotoxic potential of gzmA has recently been questioned [3], [4]. Most of what we know about granzyme function originates from in vitro studies, using purified enzymes and their delivery via perforin or other membrane-permeabilizing agents. Notably, NK cells and Tc cells from mice deficient in gzmA or B, or those defective in both gzmA and part of the gzmB cluster [5], are still capable of inducing cell death in traditional cytotoxicity assays, although their ability to induce nucleolysis and certain apoptotic features in target cells is impaired or delayed [6]. More recently, human NK cells were proposed to preferentially use gzmB to kill their targets in vitro [7]. Our knowledge of gzm function in vivo is limited. Despite the long-held assumption of gzms as the main agents of cell death induction delivered by perforin, there are relatively few in vivo models where gzmA and B play a decisive role in recovery from pathogen infection or tumour burden. Thus, mice deficient in gzmA, gzmB cluster, or both are more susceptible to infection with herpesvirus, particularly cytomegalovirus [8]–[10], and mousepox, ectromelia virus [11], [12], but their role in NK cell-mediated tumour rejection has been controversial [13]–[15]. They appear, however, to play a role in NK cell-mediated immunopathology [16]. Importantly, previous studies on the role of gzm on NK cell-mediated tumor rejection in vivo, measured long-term survival of the tumor after injection into naïve (or tumor-primed) mice, whereas the immediate pathways by which gzmA and gzmB mediate their effect in these models are still uncharacterised. Recent evidence suggest that gzmA is not cytotoxic [3], [4], but is a modulator of inflammation [3]. We have previously described that, in contrast to in vitro studies, gzmB and gzmA were not necessary for the in vivo elimination of cognate, MHC-I-restricted Tc cell targets [17]. In order to assess whether gzmA and/or gzmB are also dispensable in vivo for NK cell cytolytic function, we have used an in vivo NK cell assay targeting surface MHC-I deficient (MHC-I^neg^) targets [18]. We found that, as for cytotoxic T cells, activated NK cells without gzmA or B are able to rapidly eliminate NK cell sensitive target cells in vivo.

## Results

### Perforin-dependent elimination of MHC-I^neg^ lymphocytes from virus-primed mice

**Figure 1:**
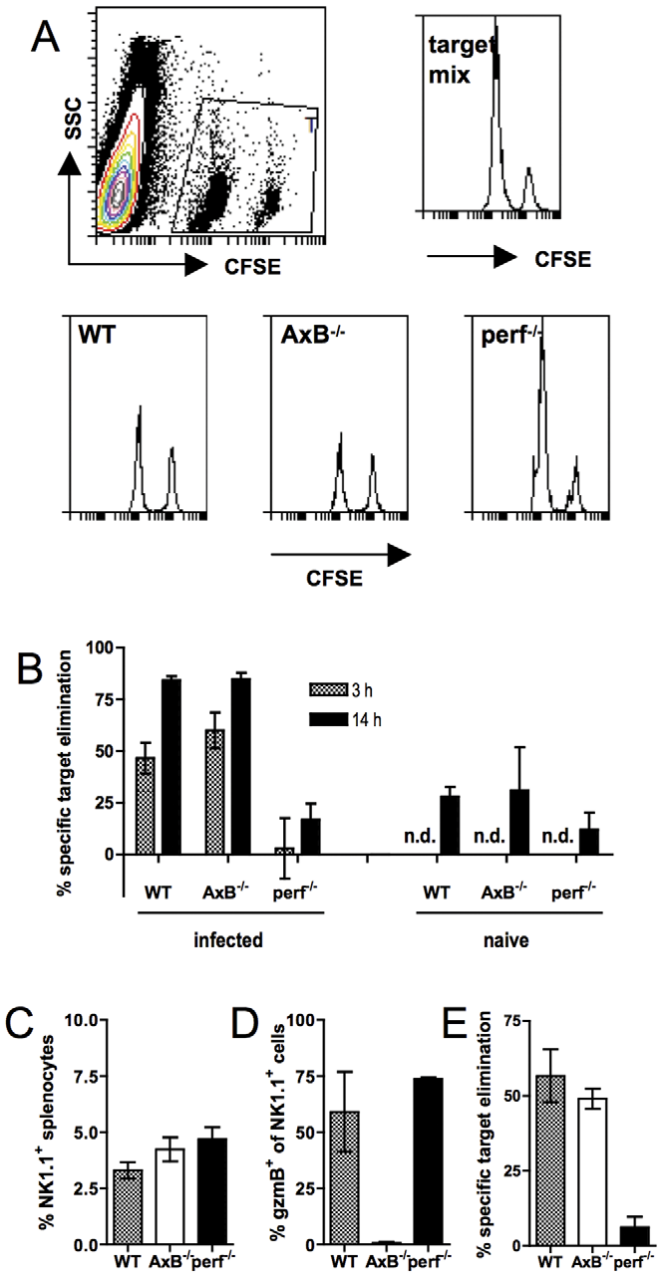
Perforin-dependent elimination of MHC-I^neg^ lymphocytes from virus-infected recipients. (A,B) Naïve or 3-day aSFV-infected B6, gzmAxB^−/−^ or perf^−/−^ mice injected i.v. with a mix of CFSE^lo^ MHC-I^neg^ and CFSE^hi^ MHC-I^pos^ splenocytes from naïve donors. Three or 14 hours after transfer, recipient spleens were analysed for the ratios of the two donor cell populations and specific elimination of MHC-I^neg^ target cells calculated. (A) Representative histograms of the donor cell ratios in the injected target mix, and in splenocyte populations recovered from WT, gzmAxB^−/−^ and perf^−/−^ recipients. (B) Comparison between specific β_2_m^−/−^ target cell elimination in WT, gzmAxB^−/−^ and perf^−/−^ recipients at 3 and 14 h post-transfer. (C,D) proportions of NK1.1^+^ splenocytes and proportions of gzmB-expressing NK1.1^+^ splenocytes. (E) Specific elimination of MHC-I^neg^ donor cells 6 hours after transfer into mice injected 36 h previously with poly-(I∶C). Data shown are means +/− STD from 3–4 mice per group, and are representative of two (E) or three (A–D) experiments. n.d. = not determined.

In order to determine short-term in vivo cytotoxicity by activated NK cells we used splenocytes from WT (MHC-I^pos^) and β_2_-microglobulin-deficient β_2_m^−/−^; MHC-I^neg^) mice as NK cell-resistant and susceptible targets, respectively [18]. We used infection with an avirulent strain of Semliki Forest virus (aSFV) to induce NK cells in vivo, because it a) induces a potent NK cell response but no antiviral CD8 T cell response in C57Bl/6 mice [16], [19], and b) does not result in pathology in gzmAxB^−/−^ or perf^−/−^ mice (data not shown). Splenic NK cell activity as a result of aSFV infection peaks three days post-infection [20]. A mixture of CFSE-labelled WT and β_2_m^−/−^ splenocytes was injected i.v. into virus-infected recipient mice that were deficient or not in various components of the granule exocytosis pathway (gzmA+B and perforin). At 3 and 14 hours after cell transfer, recipient spleens were removed and the donor target cells (which were detectable by virtue of their CFSE-mediated fluorescence) enumerated by flow cytometry, and the specific elimination of β_2_m^−/−^ splenocytes (specific target cells) relative to WT splenocytes (control cells) calculated, comparing the observed ratio of the two cell populations in the recovered cell populations and their ratio in the injected mixture. Three hours after transfer, about half or more of the MHC-I^neg^ target cells had disappeared from the spleens of WT and gzmAxB^−/−^ recipients, whereas in perf^−/−^ recipients the ratio of MHC^pos^ to MHC-I^neg^ targets was virtually the same as that originally injected (Fig. 1A,B). By 14 hours post-transfer, MHC- target cell clearance had further advanced in WT and gzmAxB^−/−^ recipients, and at this point even infected perf^−/−^, as well as naïve, recipients now also showed significant, albeit much lower, specific target cell elimination. Similar ratios, but much lower total numbers, of control to specific targets were found in lymph nodes (data not shown). This target cell elimination was not unique to SFV-infected mice, but also found in mice that were infected with influenza virus (data not shown) or mice treated 36 h previously with polyinosinic polycytosinic acid (poly-(I∶C)), a synthetic double-stranded RNA analogue and Toll-like receptor 3 ligand that induces potent NK cell cytotoxicity (Fig. 1E).

As reported by Ley and colleagues [8], most resting NK cells in naïve mice already expressed gzmA protein (88+/−3% of splenic NK cells and 55+/−2% of popliteal lymph node NK cells) but few expressed gzmB protein (<10% of NK cells in either spleen or popliteal lymph nodes). Three days after infection with aSFV the percentage of gzmA expressing NK cells had not changed significantly (data not shown) whereas a large (>60%), similar proportion of NK cells in gzm-sufficient animals were activated, as assessed by intracellular gzmB protein expression of NK cells in spleens (Fig. 1D) and popliteal lymph nodes (data not shown). Furthermore, GKO mice contained similar proportions of NK1.1^+^ cells (Fig. 1C), as well as similar numbers of total splenocytes (data not shown), both observations together suggesting similar “effector-to-target” ratios in vivo.

We were wondering whether gzmAxB^−/−^ mice might express other – so-called orphan – granzymes that might compensate for the loss of the supposedly principal cytotoxic gzmB, but this appeared not to be the case. If anything, gzmAxB−/− expressed lower amounts of orphan gzm mRNA than WT and perf^−/−^ mice (Table 1).

**Table 1:** Expression of cytolytic effector protein genes^a^.

	perf	gzm							
		A	B	C	D	E	F	K	M
WT	+++	+++	+++	++	−	−	+	++	++
gzmAxB^−/−^	+++	−	−	+	−	−	−	++	++
perf^−/−^	−	+++	+++	++	−	−	+	++	++

a Normalized to GAPDH gene expression (GAPDH = 1): −, <10^−5^; + 10^−5^–10^−3^; ++, 10^−3^–10^−1^; +++, >10^−1^.

### In vivo elimination of MHC-I^neg^ NK cell targets is independent of gzmA and B

**Figure 2:**
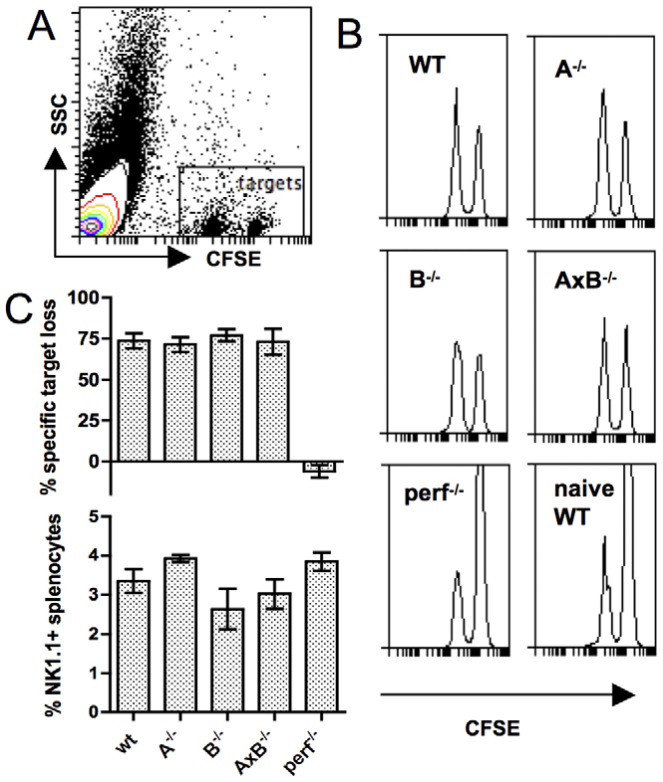
In vivo elimination of MHC-I^neg^ lymphocytes from virus-infected recipients is independent of gzmA and gzmB. Naïve or 3-day aSFV-infected single or double GKO mice were injected i.v. with a 4∶1 mix of CFSE^hi^ MHC-I^neg^ and CFSE^lo^ MHC-I^pos^ naïve splenocytes. Four hours after transfer, recipient spleens were analysed for the ratios of the two donor cell populations and specific elimination of MHC-I^neg^ target cells calculated. (A) Dot plot showing the gating of donor cells in the recipient splenocyte population. (B) Representative dot plots from infected WT , gzmA^−/−^, gzmB^−/−^, gzmAxB^−/−^, perf^−/−^, and naïve WT recipients. (C) Specific target cell elimination and proportions of NK1.1^+^ cells in recipient spleens. The data shown are means +/− SD from 3 mice per group and are representative of two experiments.

To assess the contribution, or lack thereof, of the two principal gzm to the elimination of MHC-I- lymphocytes in vivo we tested single- gzmGKO mice along with WT and perf^−/−^ mice, with similar results: over 6 hours, virus-infected WT, gzmA^−/−^, gzmB^−/−^ and gzmAxB^−/−^ mice all cleared MHC-I^neg^ lymphocytes with similar efficiency, whereas perf^−/−^ mice were severely deficient in this clearance, with the target cell ratios in virus-primed perf^−/−^ recipients resembling those found in naïve WT recipients (Fig. 2). All mice had similar proportions of NK1.1^+^ splenocytes (Fig. 2C). In some experiments, perf^−/−^ mice had up to 1.5-fold greater numbers of total NK1.1^+^ splenocytes, due to their larger overall spleen cellularity at 8 weeks of age, compared with mice of the other strains (data not shown). However, this increased number of NK cells clearly did not compensate for the absence of perforin.

### NK cell depletion abolishes the elimination of MHC-I^neg^ lymphocytes in virus-infected mice

**Figure 3:**
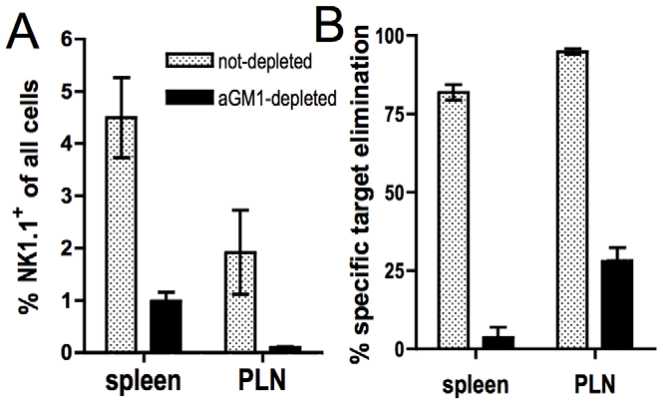
In vivo elimination of MHC-I^neg^ lymphocytes is NK-cell dependent. GzmAxB^−/−^ mice were infected with aSFV and injected at days 2 and 3 with anti-asialo GM1 antibody. At day 3 post-infection, CFSE-labelled target cells were also injected and target cell elimination analysed after 4 hours as in Fig. 1. (A) Proportion of NK1.1^+^ cells in spleen and popliteal lymph nodes of recipient mice after sacrifice. (B) Specific elimination of MHC-I^neg^ target cells in NK-depleted or non-depleted mice. Data shown are means +/− SD.

Although Öberg et al. already demonstrated in their studies that rejection of β_2_m^−/−^ lymphocytes in this adoptive transfer model was mediated by NK cells, their assays were based on naïve recipients. To ascertain that this was also the case in mice with virus-activated NK cells, we depleted NK cells in our virus-infected recipients by in vivo administration of anti asialo-GM1 antibody and performed the aforementioned in vivo transfer assays. Depletion of NK1.1^+^ cells resulted in a substantial loss of the recipients' ability to reject MHC-I^neg^ targets (Fig. 3), demonstrating that in this model, too, target cell killing is mediated by NK cells. CD8+ T cells were unaffected (data not shown) although it is unlikely that they contributed to the observed target cell clearance, since a) aSFV does not generate CD8 T cell responses in C57BL/6 mice [16], and b) the recipients' CD8^+^ cells were only displaying a partially activated phenotype (CD69^+^CD25^−^, [21]).

### GzmA- and gzmB-independent, rapid rejection of MHC-I- targets in virus-infected mice

**Figure 4:**
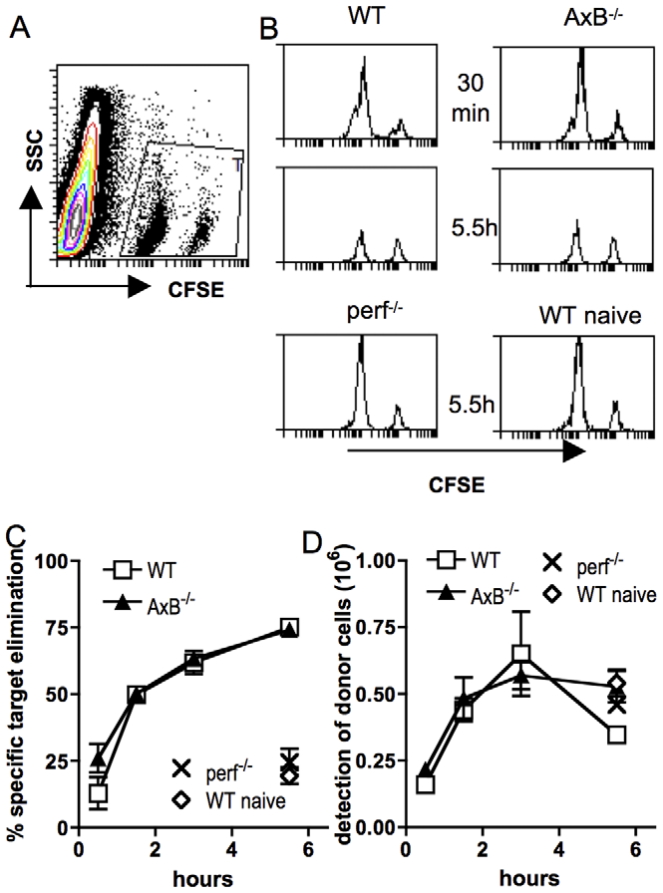
In vivo elimination of MHC-I^neg^ lymphocytes in WT and gzmAxB^−/−^ is equally rapid. 3-day aSFV-infected or naive mice of the shown genotype were injected with 1∶5 mix of CFSE^lo^ MHC-I^neg^ and CFSE^hi^ MHC-I^pos^ naïve donor splenocytes. (A) Dot plot showing the gating of donor cells in the recipient splenocyte population. (B) Representative histograms gated on donor cells in infected WT, gzmAxB^−/−^, perf^−/−^ or naïve WT mice after 30 minutes or 5.5 h. (C) Specific elimination of MHC-I^neg^ donor cells at various time points. (D) Tracking of total WT control donor cells in WT gzmAxB^−/−^ and perf^−/−^ recipients. Data shown are means +/− SD from 3–4 mice per group and are representative of three experiments.

We have shown that virus-immune Tc cells do not require gzmA and gzmB for very rapid in vivo target cell killing, with the majority of targets eliminated by less than 45 minutes after transfer [17]. We therefore assessed the kinetics of NK cell-mediated rejection of MHC-I- lymphocytes in vivo. We found that WT recipients cleared the majority of target cells by 90 minutes, with significant killing already demonstrable 30 minutes after transfer, and clearance reaching 75% of MHC-I^neg^ targets by less than 6 hours (Fig. 4A–C). Importantly, gzmAxB^−/−^ displayed identical kinetics of target cell clearance, suggesting that these granzymes are not required for the rapid in vivo killing kinetics observed in virus-infected mice. Although both virus-infected perf^−/−^ as well as naïve WT recipients showed significant target elimination by 6 hours, the observed levels were similar only to those observed after 30 minutes in infected WT recipients.

Although WT C57Bl/6 and gzmAxB^−/−^ mice have similar organogenesis and lymphoid compartments [6], and similar proportions and numbers of NK cells were found in both strains, we further assessed whether there were differences in the migration of adoptively transferred donor cells to the recipients lymphoid organs. Although control target cell recovery varied in efficiency between experiments, within individual experiments we always found WT control donor target cells in similar proportions and numbers in both spleens (Fig. 4D) and popliteal lymph nodes (data not shown), suggesting that these features are also unaltered by the genetic alterations in the GKO strain.

## Discussion

We have used an assay measuring the NK cell-mediated elimination of MHC-I^neg^ lymphocytes in vivo to test the requirement for the two principal gzm of cytolytic lymphocytes, gzmA and gzmB, in this process. We show that rapid elimination of target cells occurs almost identically in virus-infected mice that are sufficient or deficient of these gzm, further challenging the previously held dogma that they are required for rapid and efficient target cell killing. Although even naïve recipients were able to reject target cells, rapid kinetics of target cell rejection, with the majority of targets eliminated by 90 minutes post-transfer, required that the mice be either virus-infected or stimulated with NK cell-inducing reagents. These results are remarkably similar to our recent findings made in a system of MHC-I-restricted, cognate antigen-specific target cell killing by antiviral Tc cells [17], thus leading to similar implications for the importance of gzmA and gzmB in both Tc cell-mediated and NK cell-mediated killing.

Given that NK cells in perf^−/−^ mice expressed gzmB at levels similar to those in WT mice, but these mice were severely deficient in rejection of MHC-I^neg^ targets, the gain of cytotoxic effector function observed after infection-mediated activation of NK cells is likely not due to the observed gain in gzmB protein expression after activation, but more likely due to the concurrent gain in perforin protein expression [8]. Injection of β_2_m^−/−^ splenocytes by itself did not result in upregulation of gzmB expression in NK cells from naïve mice (data not shown). However, it was possible that the gzmAxB-independent but perf-dependent in vivo cytotoxicity by NK cells reported here was mediated by granzymes other than A and B, which might be expressed in gzmAxB^−/−^ mice as a compensatory mechanism. Such a redundancy has been proposed as an explanation for the lack of clear-cut phenotypes associated with single gzm KO mice [22], [23]. However, we found that only gzmC was expressed differentially, with higher expression in WT and perf^−/−^ cells than in gzmAxB^−/−^ cells (Table 1), a finding consistent with the “knock-down” effect described for gzm genes downstream of gzmB in the gzmB^−/−^ strain [5], from which the gzmAxB^−/−^ mice were bred. An increased gzmC expression was also observed in Tc cells from virus-infected mice of that strain [17]. Thus, we did not observe any differential gzm gene expression in gzmAxB^−/−^ NK cells that might have compensated for the loss of the postulated cytotoxic requirement for gzmA and gzmB. However, a difference in orphan gzm protein expression cannot formally be excluded since, as has been shown for gzmB, mRNA expression does not necessarily correlate with protein expression [8]. It is also highly unlikely that NK cytolytic function in the gzm deficient mouse strain was mediated via the Fas pathway because if it were, one would expect such Fas mediated redundancy also to be observed in perf^−/−^ mice.

While our data question an important in vivo role for gzmA and gzmB in the rejection of naive MHC-I^neg^ lymphocytes, there may be other situations where target cell rejection in vivo by NK cells is mediated by other pathways. Notably, Grundy et al demonstrated that tumor cells locating to the lung after injection were rejected, also with quite rapid kinetics, but via a perforin-independent pathway [24]. Also, injection of YAC-1 and RMA-S cells, targets traditionally used as NK cell targets in vitro, led to rapid, NK-dependent elimination of these cells in the recipient lungs [25], although the dependency of this process on perforin was not established in that study. It therefore appears, just like for in vitro apoptosis assays [26], that the nature of the target cell has a profound influence on the cytotoxic pathways employed by cytolytic lymphocytes. Arguably, one would expect transformed tumor cells to express a range of NK cell activating signals, whereas the only signal in β_2_m^−/−^ is the loss of MHC-I. An important caveat of the type of assay used here is that only a small proportion of injected donor splenocytes are recovered from the recipient spleens at any one time (typically less than 10%; [17] and data not shown) and it is conceivable that a large proportion of cells suffers an uncertain fate elsewhere in the receipient. However, despite similar limitations, this technique has been found useful and widely applied to investigate in vivo Tc cell cytotoxicity [27].

In order to broaden our target cell range in our investigations, we also used mixtures of (MHC-I^pos^) RMA and (MHC-I^neg^) RMA-S cells. However, these cells do not localize to the spleen after i.v. injection, and both the proportion as well as absolute number of NK cells in the putatively relevant compartments (lungs and peritoneum, after i.v. or intraperitoneal target cell injection, respectively) were substantially lower in gzmAxB^−/−^ than in WT recipients, rendering analysis of the data difficult due to the varying effector to target cell ratios. The observation of reduced NK cell migration in the absence of gzm is consistent with the notion that these enzymes have a role in lymphocyte homing and migration ([28], [29], and Murphy et al., manuscript in publication). In both these models, however, the magnitude of target cell rejection correlated well with local NK cell presence and perf-WT genotype, but not with gzm expression (data not shown).

In conclusion, we show that, as for target cell elimination by MHC-I-restricted Tc cells, rapid elimination of MHC-I^neg^ lymphocytes by NK cells in vivo is perforin-dependent while requiring neither gzmA nor gzmB. Together with our observations for Tc cell-mediated in vivo cytotoxicity, [17], these findings add weight to the notion that at least murine gzmA may not be cytotoxic, while the cytotoxic potential of gzmB is still maintained by others [3], [4]. Further research into their in vivo function are required to elucidate whether either gzm performs hitherto unknown functions.

## Materials and Methods

### Mice and virus

Female C57BL/6 and gene-KO mice were obtained, at 6–8 weeks of age, from the specific pathogen-free facility at the John Curtin School of Medical Research, and used according to protocols approved by the Animal Experimentation Ethics Committee (J.IG.68.08, J.IG.51.06, J.IG.19.03). GzmAxB^−/−^ mice were originally generated by crossing the gzmA^−/−^ [22] and gzmB^−/−^ [30] mice also used in this study. Perf^−/−^ mice were originally generated by Kägi et al. [31]. aSFV [20] and influenza A/PR8 viruses [32] were grown and assayed as described previously. Genotype of mice used in experiments was confirmed by PCR.

### In vivo killing assays

For in vivo NK cell assays mice were infected i.v. with 10^7^ PFU aSFV, or 10^3^ haemagglutination forming units influenza A/PR8, or injected i.p. with 100 µg poly-(I∶C). Three days after virus infection or 36 h after poly-(I∶C) injection, mice received 2×10^7^ cells of a mixture of CFSE labelled control WT or β_2_m^−/−^ splenocytes i.v.. The cell ratios and CFSE concentrations used varied between experiments. Typically, a ∼4∶1 ratio of cognate to control cells were used in order to better follow the loss of cognate target cells, but results were similar when equal ratios were used. CFSE concentrations were 200–400 nM and 4–5 µM for the CFSE^lo^ and CFSE^hi^ target cells, respectively. At the indicated time points after transfer, recipient spleens and popliteal lymph nodes were harvested and donor target cells enumerated by FACS based on their CFSE profiles.

### Flow cytometry

Red-blood cell-lysed splenocytes, lung, lymph node and peritoneal exudate cells were incubated in 7-aminoactinomycin D and stained with antibodies against NK1.1 (both Pharmingen). Cells were then fixed in 2% fresh paraformaldehyde, permeabilized with 0.5% saponin and stained with polyclonal rabbit anti-gzmA or anti-gzmB antiserum, followed by Alexa647-conjugated rat anti-rabbit IgG (Molecular Probes). Cells were then analysed on a FACSCalibur (Becton Dickinson). % Specific target cell loss was calculated as % SL = 100–100/(number of recovered control cells)*(number of recovered specific target cells)*(ratio control∶specific target cells in injected mix).

### Analysis of perf and gzm gene expression

Total mRNA was isolated from 5×10^5^ NK1.1^+^ cells, positively enriched by autoMACS (Miltenyi, Germany; according to the manufacturer's protocol), reverse-transcribed and assayed with TaqMan Gene Expression assays (Applied Biosystems) on a 7900HT Fast real-time PCR system (Applied Biosystems, at the ACRF Biomolecular Resource Facility, JCSMR). The copy number obtained for each gene of interest was normalized to the copy number of GAPDH mRNA ( = 1).
